# Diagnosis and management of adenosine deaminase 2 deficiency children: the experience from China

**DOI:** 10.1186/s12969-021-00535-z

**Published:** 2021-03-23

**Authors:** Wei Wang, Tiannan Zhang, Wenjie Zheng, Linqing Zhong, Lin Wang, Ji Li, Qian Liu, Yanqing Dong, Hongmei Song

**Affiliations:** 1 Department of Pediatrics, Peking Union Medical College Hospital, Chinese Academy of Medical Sciences, Beijing, China; 2 Department of Rheumatology, The Second Affiliated Hospital and Yuying Children’s Hospital of Wenzhou Medical University, Wenzhou, Zhejiang Province China; 3 Department of Clinical Laboratory, Peking Union Medical College Hospital, Chinese Academy of Medical Sciences, Beijing, China

**Keywords:** Adenosine deaminase 2, Autoinflammatory disease, Whole-exome sequencing, China, Hematopoietic stem cell transplantation

## Abstract

**Background:**

Deficiency of adenosine deaminase 2 (DADA2) is a rare autoinflammatory disease caused by mutations in the ADA2 gene. Few Chinese cases have been reported. We describe and compare the clinical features, genotypes, and treatments of Chinese DADA2 patients and non-Chinese patients.

**Methods:**

Primary immunodeficiency disease panel or whole-exome sequencing was performed for suspected cases, and assays for adenosine deaminase 2 (ADA2) enzyme activity were also carried out for the patients and their parents. Case reports of Chinese and non-Chinese patients with DADA2 were searched in PubMed and Chinese national databases.

**Results:**

Seven unrelated children from China with DADA2 were included in our study. Five were identified at Peking Union Medical College Hospital, and two had been reported previously (1 on PubMed and 1 in Chinese literature). Fourteen mutations in ADA2 were identified, 7 of which have not previously been reported in non-Chinese patients. Four children who underwent enzymatic analysis had lower ADA2 activity compared with their parents. Phenotypic manifestations included fever, skin symptoms, vasculitis, and neurologic involvement. Treatments varying from steroids, immunosuppressants, and tocilizumab, anti-TNF therapy and hematopoietic stem cell transplantation (HSCT) were effective depending on phenotype and severity.

**Conclusion:**

This study includes the largest number of Chinese DADA2 patients to date. We recommend the combination of enzymatic analysis with gene screening to confirm the diagnosis. Different genotypes were observed among Chinese DADA2 patients; most phenotypes were similar to those of non-Chinese DADA2 patients, except for growth retardation. Disease remission might not be achieved with anti-IL-6 therapy.

## Background

Deficiency of adenosine deaminase 2 (DADA2) is a rare, autosomal recessive autoinflammatory disease that is caused by mutations in the ADA2 gene [1]. DADA2 is usually a childhood-onset disease, with 24% of cases reported before 1 year of age, and 77% before the age of 10. DADA2 was first described in 2014 [1, 2], and more than 300 cases have been reported in the literature thus far [3–7]. ADA2 is secreted by monocytes and macrophages, and may have a role in the degradation of extracellular adenosine at the site of inflammation. Minimal residual activity of ADA2 enzyme was thought to be associated with pure red cell aplasia and bone marrow failure [4]. Several reports have also suggested that DADA2 may cause broad-spectrum manifestations, including early-onset stroke, nodular vasculitis, immunodeficiency, red-cell aplasia, and neutropenia. However, some reports have shown no evidence for vasculitis in patients with DADA2 [8], with hematologic involvement or lymphoproliferation possibly being the only manifestation [9].

Mutation sites seem to vary among patients in different parts of the world. For instance, the most frequent mutations are G47R in Turkish and Georgian ancestry, and R169Q in the European population [9–11]. The genotype-phenotype correlation has been studied, linking the G47R mutation with a poor prognosis and the PAN-like phenotype, and the R169Q mutation with stroke. Treatment response may also differ due to the variable phenotypes. Unlike patients with DADA2 and vasculitis, cases with pure red cell aplasia and bone marrow failure are largely refractory to TNF inhibitors [12]. It remains unclear whether ethnicity plays a role in disease expressivity and therapy effectiveness. Reports from China are rare but needed. In this study, we evaluated 7 Chinese children with DADA2 to assess Chinese features of DADA2 with regard to clinical manifestation, genotype, therapeutic response, and outcome.

## Methods

### Patients

Patients from Peking Union Medical College Hospital (PUMCH) suspected of having DADA2 were enrolled since 2016. The inclusion criteria in this study were (1) early-onset livedo reticularis associated with chronic or recurrent signs of systemic inflammation, (2) hemorrhagic/ischemic stroke, or signs of peripheral nervous system involvement associated with systemic inflammation, and/or (3) previous diagnosis with childhood-onset PAN. (4) The diagnosis was made depending on gene analysis. The ethics committees of the PUMCH approved the study(ZJS-1248, JS-1660).

### Genetic analysis

Blood samples of suspected children and their parents were collected after consent was obtained. Genomic DNA was extracted by standard methods. To identify candidate genes and mutations, gene capture high-throughput sequencing with primary immunodeficiency disease (PID) panel was performed between 2016 and 2017; whole exome sequencing was performed since 2018. Bioinformatics analysis was conducted as follows. Variations with MAF > 0.01 or located further than 10 bp from exons were filtered out. The pathogenicity of the candidate site was assessed using multiple software such as SIFT, Polyphen, Mutation Taster. Benign or likely benign variations were eliminated, retaining pathogenic and, likely pathogenic variations, and those with uncertain significance. The data were then combined with patients’ clinical features to select candidate genes related to the disease. Finally, the candidate mutations were verified by Sanger sequencing.

### ADA2 activity detection

The activity of total ADA and ADA2 in the serum of suspected patients with ADA2 mutations and their parents was assayed. Peripheral blood was taken from children suspected of having DADA2. Serum was separated by centrifugation; total ADA activity was detected by an automatic biochemical analyzer, and ADA2 activity was detected by inhibiting ADA1 activity with EHNA (final concentration of the reaction was 40 μmol/L). The parents were used as control. There was one patient whose ADA2 activity was first detected by ELISA at a local hospital. One unit of ADA activity was defined as the μmol amount of hypoxanthine nucleosides produced from adenosine per minute at 37 °C.

### Clinical data collection

All subjects with the diagnosis of DADA2 were evaluated, and respective data from patient files, including previous diagnosis, comprehensive and retrospective clinical course, imaging, and laboratory results, were recorded. Medications used in the treatment before and after the diagnosis of DADA2 were also collected.

### Search strategy

We used PubMed, Chinese database “CNKI”, and “WanFangData” together with “CQVIP” to search for literature written in English and Chinese. The terms included “Deficiency of adenosine deaminase type 2”, “adenosine deaminase type 2 deficiency”, “ADA2 deficiency”, “deficiency of ADA2” and “DADA2”. We examined all studies published up to June 2020. Cases from China were included, and non-Chinese cases were used for comparison.

### Statistical analysis

Fisher’s exact test was used to compare the difference in the rate of abnormal manifestations and abnormal library test ratio between Chinese and non-Chinese patients. Statistical software was SPSS version 22.0. The threshold for statistical significance was set to *p* < 0.05.

## Results

### Patients and genetics

A total of 16 patients suspected with DADA2 had investigated records. Five of them were diagnosed with DADA2 followed in the Department of Pediatrics, PUMCH, one of whom was reported in the Chinese literature in October 2018. In addition, 1 case was described in the Chinese database, from the Capital Institute of Pediatrics, China in July 2019 [13]. For PubMed, more than 300 patients have been reported to date, among whom the only one was from Children’s Hospital of Fudan University, China [14].

We identified compound heterozygous mutations in ADA2 in 5 patients from PUMCH (Table 1). Although only one heterozygous variation was initially found in P2, his decreasing activity of ADA2 indicated that he had DADA2. Thus, we reanalyzed his primary sequencing data using IGV (integrated genome viewer) and checked the read numbers of ADA2 to determine the presence of any large deletions. Finally, deletions of exon 7 was confirmed as a maternal deletion mutation by qPCR. The other 2 cases reported previously carried 2 compound heterozygous mutations each.

**Table 1:** Pathogenic variants in patients with DADA2 from PUMCH

		P1	P1	P2	P3	P3	P4	P4	P5	P5
ADA2 mutation	Nucleotide Changes	c.1232A > G	c.983A > T	c.13G > C	c.878A > C	c.263A > G	c.506G > A	c.393delG	c.254A > T	c.851G > T
	Amino Acid Changes	p.Y411C	p.N328I	p.G5R	p.H293P	p.Y88C	p.R169Q	p.R131Sfs^b^52	p.N85I	p.G284V
	Gene Type	het	het	het	het	het	het	het	het	het
Gene Database	dbsnp147	rs376785840	–	rs202134424	rs2231495	–	rs77563738	–	rs547716532	–
	PathSNP	N/A	N/A	N/A	N/A	N/A	N/A	N/A	N/A	N/A
	MutInNormal	N/A	N/A	N/A	N/A	N/A	N/A	N/A	N/A	N/A
	1000Genome	N/A	N/A	N/A	chr22–17,669,306	N/A	chr22–17,687,997	N/A	N/A	N/A
	MutInDatabase	N/A	N/A	N/A	N/A	N/A	N/A	N/A	N/A	N/A
MAF	1000g2015aug_all	–	–	–	–	–	0.0002	–	–	–
	ESP6500si	0.0002	–	–	–	–	0.0005	–	–	–
	ExAC_ALL	0.0001	–	0.0000	0.00002478	–	0.0005	–	0.0000	–
	ExAC_EAS	–	–	–	–	–	–	–	–	–
Patho-genicity speculated by software	SIFT	0.003	0.0	0.002	0.2	0.002	0.0	–	0.001	0.003
	SIFT_Predict	Damaging	Damaging	Damaging	Tolerated	Damaging	Damaging	–	Damaging	Damaging
	PolyPhen2	0.998	1.0	0.999	0.055	0.999	0.993	–	0.973	1.0
	PolyPhen2 Predict	Probably damaging	Probably damaging	Probably damaging	Benign	Probably damaging	Probably damaging	–	Probably damaging	Probably damaging
	MutationTaster	0.999	1.000	1	1	1	1	–	1.000	1
	MutationTaster Predict	Disease causing	Disease causing	Disease causing	Polymorphism_automatic	Disease causing	Polymorphism	–	Disease causing	Disease causing
	GERP++	2.73	2.68	4.76	0.283	3.26	2.21	–	3.54	4.02
	GERP++ Predict	Conserved	Conserved	Conserved	Nonconserved	Conserved	Conserved	–	Conserved	Conserved

N/A, not available

The 7 Chinese children were from 7 unrelated families, all born to nonconsanguineous parents. There were 14 mutation sites, including 1 deleted exon, 1 frameshift mutation and 12 missense mutations. Seven mutations have been reported previously in the other countries; 7 are novel mutations only found in Chinese. ADA2 activity was measured in 4 patients (3 cases were tested by automatic biochemical analyzer at PUMCH; P3 was assessed by ELISA at a local hospital), and was significantly reduced compared to the levels in the parents’ (Table 2).

**Table 2:** Summary of clinical characteristics, treatment, and genotype in the 7 subjects with ADA2 deficiency from China

	P1	P2	P3	P4	P5	P6 [13]	P7 [14]
Gender	female	male	male	male	female	male	female
Age at onset/diagnosis	3y/6y	9y/16y	2 m/6y	6 m/1y	1y/5y	4y/8y	3 m/13 m
CECR1 mutations							
Paternal	c.1232A > G, p.Y411C^a^	c.13G > C, p.G5R^a^	c.878A > C, p.H293P^a^	c.506G > A, p.R169Q^a^	c.254A > T, p.N85I^a^	c.1072G > A, p.G358R	c.1211 T > C, p.F404S
Maternal	c.983A > T, p.N328I	Exon7del^a^	c.263A > G, p.Y88C	c.393delG, p.R131Sfs^b^52	c.851G > T, p.G284V	c.142G > A, p.G48R	c.1114G > A, p.V372M
ADA2(U/L)	Not tested	0	0.67^b^	0.2	0	Not tested	Not tested
ADA2(U/L) of Father/Mother	Not tested	Not tested / 1.58	5.86/3.01^b^	1.8/3.44	1.91/2.17	Not tested	Not tested
total ADA(U/L)	Not tested	3.45	Unscanned	3.64	3.61	Not tested	Not tested
Fever/Age at onset	Yes/3y	Yes/14y	Yes/2 m	Yes/6 m	Yes/2y	YES/4y	Yes/3 m
Skin symptom	Yes/3y	Yes/9y	Yes/2 m	Yes/7 m	Yes/2y	YES/4y	Yes/3 m
	Erythema (on both lower limbs)	Erythema (Hip, all four limbs)	Erythematous papule (on face)	Livedo reticularis (on both lower limbs)	Erythema nodosum and Livedo reticularis (on both lower limbs)	Erythema nodosum (face and all four limbs) Livedo reticularis (on left lower limbs)	Erythema (on both lower limbs) Necrosis (on upper extremities) upper extremities
Neurological involvement	No	No	No	No	2y/ hemorrhagic stroke at left basal ganglia Eye external oblique (right eye), abnormal gait	4y/ ischemic stroke blepharoptosis (both eyes), Eye external oblique, abnormal gait	1y/ ischemic stroke at right thalamus blurred vision (right eye)
Abdominal pain	Yes/3y	Yes/9y	No	No	Yes/5y	No	No
Nephrological involvement	No	Unilateral Hydronephrosis(left)	Hypertension and Microalbuminuria	No	No	No	No
Arthritis	No	No	No	No	2y/ bilateral ankle joint	4y/ bilateral ankle joint	No
Growth retardation	No	No	No	YES/1y2m Wt 7.6 kg(<P3) Ht 67.5 cm(<P3)	No	No	YES/1y (unspecified)
Oral aphthous	No	No	No	No	Yes/2y	No	No
WBC(× 10^9^/L)	4.14	4.79	9.06	21	15.51	7	N/A
HGB (g/L)	122	124	120	98	132	90	Anemia (unspecified)
PLT(×10^9^/L)	224	118	429	132	392	413	454
CRP (mg/L)	3	11	80	43	115	59	49.98
ESR (mm/h)	9	6	95	49	74	16	N/A
IL-6 (pg/ml)	N/A	24.9	Not tested	33.3	230	N/A	120.85
IL-8 (pg/ml)	N/A	N/A	Not tested	11	27	N/A	135.25
TNF-a (pg/ml)	N/A	16.6	Not tested	22.8	41.6	23.3	2.79
IgG (g/L)	5.33	6.84	1.92↓	6.95	6.81	Low(unspecified)	10.24
IgA (g/L)	0.46	0.95	0.31	0.66	0.41	N/A	0.58
IgM (g/L)	0.31↓	0.23↓	0.17↓	1.07	0.56	N/A	0.72
IgE (KU/L)	36.3	64.5	27.14	24	Unscanned	N/A	N/A
ANA	Negative	Negative	6y/ANA 1:100; SSA/AMA (±)	Negative	Negative	N/A	N/A
CD3%	70.5%	76.8%	69.3%	22%	84.5%	N/A	51.8%
CD4%	42.2%	28.3%	28.0%	14%	49.4%	N/A	30.05%
CD8%	21.7%	40.6%	28.4%	7.4%	31.6%	N/A	18.45%
B%	23.5%	16.3%	24.8%	65%	8.3%	N/A	26.49%
NK%	5.6%	4.8%	5.8%	10.7%	6.5%	N/A	20.63%
Previous treatment	–	CS, MTX	CS, IVIG	CS, IVIG	CS, Colchicine, IVIG, TCZ	CS, MTX, CTX	CS, TCZ
Current treatment	Dipyridamole	MMF	MTX	Adalimumab	Etanercept	Anti TNF-α (unspecified)	HSCT

^a^, reported previously in non-Chinese DADA2 patients; ^b^, tested by ELISA at local hospital

Reference values for immunoglobulins (aged 1-6y): IgG 5-12 g/L, IgA 0.2–1.08 g/L, IgM 0.5–1.99 g/L

*N/A* Not available

*CS* Corticosteroid, *CTX* Cyclophosphamide, *IVIG* Intravenous Ig, *NSAID* Nonsteroidal anti-inflammatory drugs, *MTX* Methotrexate, *MMF* Mycophenolate mofetil, *TCZ* Tocilizumab, *HSCT* Hematopoietic stem cell transplantation

### Clinical features and laboratory testing

The median age at presentation was 2 years of age (range 2 months to 9 years of age). All patients underwent thorough clinical and laboratory investigations in local hospitals, excluding genetic evaluation, before their referral. The median time from the onset of symptoms to confirmed diagnosis was approximately 41 months (range, 6 months to 7 years). The mean follow-up time was 10 months (range, 7 to 15 months).

**Fig. 1:**
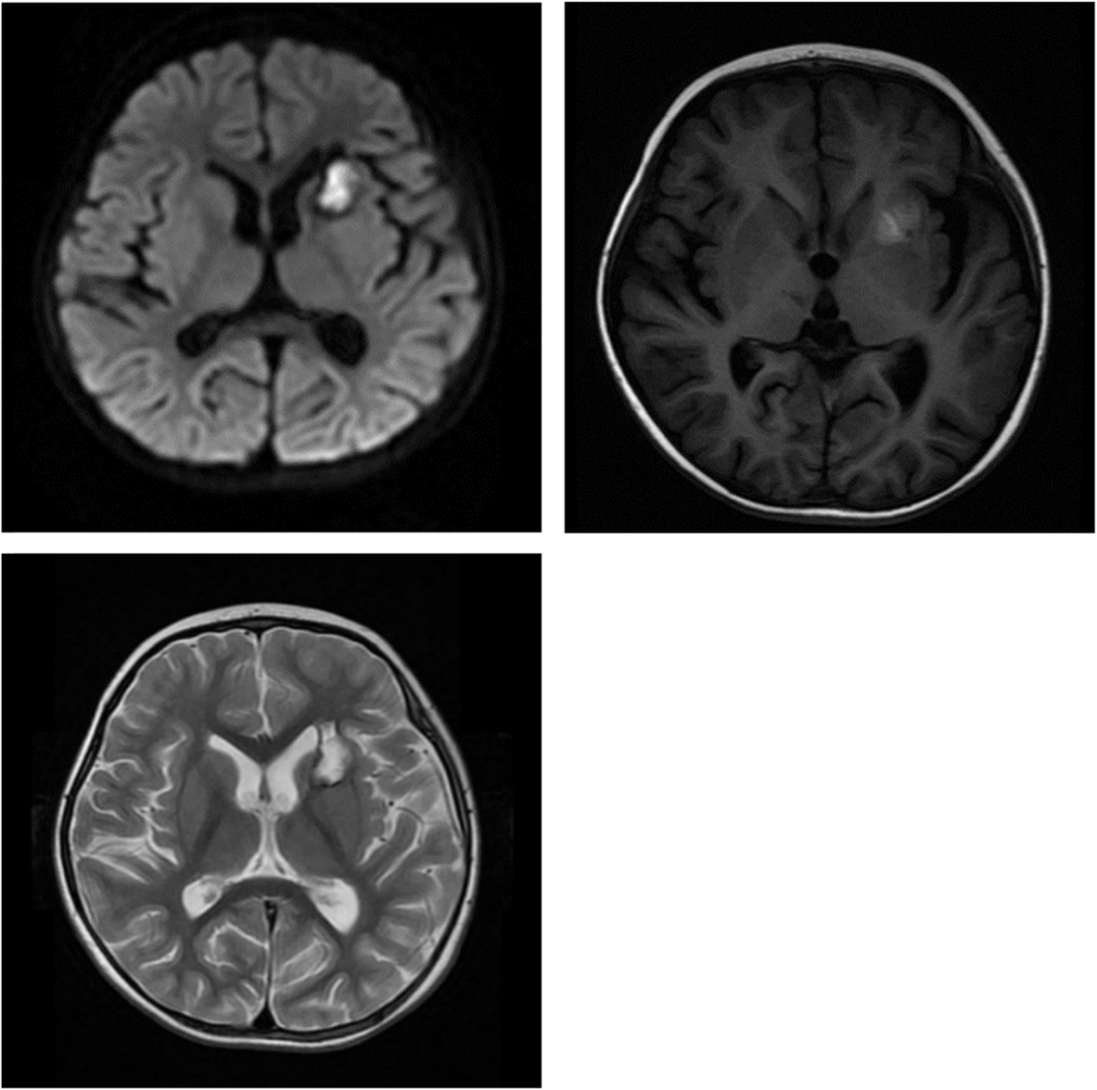
Hemorrhagic stroke at left basal ganglia seen on cerebral magnetic resonance image (MRI)

All patients presented with fever and rash. Six patients had erythema and livedo reticularis located on the lower limbs, and one had erythematous papule on his face. Only one patient presented with digital necrosis of the fingers; 3 patients experienced early-onset stroke (1 hemorrhagic and 2 ischemic, Fig. 1 and Table 2), all accompanied by eye dysfunction (2 with eye external oblique, and 1 with blurred vision). Two patients also had an abnormal gait. Nephrological manifestations were detected in 2 patients, including unilateral hydronephrosis and hypertension together with microalbuminuria. Three of them experienced abdominal pain. None of our patients had hepatosplenomegaly. Bilateral ankle joint arthritis was present in 2 patients. In addition, 2 patients were found to have growth retardation, as their weight and height were lower than 3% in the same age group. None of the patients displayed a clear history consistent with recurrent infections.

Regarding laboratory tests, mild anemia was detected in 2 patients. Acute phase reactants were elevated in all but one patient. Elevated IL-6 was found in 2 patients. Three patients had lower levels of IgM, and 2 had a lower level of IgG. P3 was the only patient who presented with ANA positicity, with a 1:10 titer and SSA/AMA suspicious; the other patients were negative. Lymphocyte subpopulation analysis was performed for 6 patients, with normal results.

### Treatment and outcomes

Six patients received corticosteroid (CS) as initial therapy, and immunosuppressive drugs, such as methotrexate (MTX) and mycophenolate mofetil (MMF), were used when tapering CS. Tocilizumab (TCZ), anti-TNF therapy, and hematopoietic stem cell transplantation (HSCT) were performed as previous treatments had failed.

P1 was treated only with dipyridamole, and her erythema as well as her fever improved. The other patients had a chronic disease course that relapsed following steroid tapering. Two patients (P2 and P3) showed a complete response, one each to MMF and MTX. Three patients (P4, P5, P6) were started in anti-TNF therapy because of the poor response to other treatment and remained in remission. It is worth mentioning that 2 patients (P5, P7) were treated with TCZ due to elevated levels of IL-6, and both showed a good response, with gradual reduction in steroid dosage. However, the remission only lasted for no more than half a year, relapse eventually ensued; P7 even experienced cerebral stroke. P7 underwent hematopoietic stem cell transplantation (HSCT) and showed substantial improvement. Currently, all these patients are stable.

### Comparison

The largest number of DADA2 patients included in one single article was 161 [3]. These patients were from Turkey, the USA, and European countries, whereas no patients were from China. In our study, the clinical features were mostly similar (Table 3). The rates of fever and abdominal pain were higher among Chinese patients. Moreover, some manifestations were not present, such as hepatosplenomegaly, neutropenia, lymphopenia, and low levels of IgA.

**Table 3:** Comparison of DADA2 patients between China and other countries

	DADA2 patients from China (*n* = 7)	DADA2 patients from Other countries ref. 2(*n* = 161)	*P* Value
Gender M: F	57%: 43%	53%: 47%	–
Onset age < 1y	3 (42%)	38 (24%)	NS
Fever	7 (100%)	81 (50%)	0.014
Skin manifestations	7 (100%)	121 (75%)	NS
Livedo reticularis	6 (86%)	81 (50%)	NS
Digital necrosis	1 (14%)	36 (22%)	NS
Aphtous ulcers	1 (14%)	11 (7%)	NS
CNS ischemic stroke	2 (29%)	43 (27%)	NS
CNS hemorrhagic stroke	1 (14%)	19 (12%)	NS
Abdominal pain	3 (43%)	19 (12%)	0.048
Splenomegaly	0 (0%)	47 (29%)	NS
Hepatomegaly	0 (0%)	31 (19%)	NS
Nephrological manifestations	2 (29%)	23 (14%)	NS
Hypertension	1 (14%)	34 (21%)	NS
Arthritis	2 (29%)	23 (14%)	NS
Neutropenia	0 (0%)	11 (7%)	NS
Anemia	2 (29%)	21 (13%)	NS
Lymphopenia	0 (0%)	26 (16%)	NS
Hypogammaglobulinemia	2 (29%)	36 (22%)	NS
Low IgM	3 (42%)	29 (18%)	NS
Low IgA	0 (0%)	19 (12%)	NS

*CNS* Central nervous system, *NS* No significance

## Discussion

Mutations in the ADA2 gene cause reduced or absent ADA activity. The identified biallelic mutations of ADA2 accompanying the clinical phenotype contribute to the diagnosis; the plasma or serum ADA2 activity test is helpful [15]. Currently (up to 2020.04), 81 mutations have been registered at HGMD. However, genotype-phenotype correlations in DADA2 have been difficult to establish because of incomplete penetrance and variable clinical manifestations in patients with identical mutations [7]. The most frequent mutation G47R, which is thought to be more associated with the PAN-like phenotype, in Turkish and Georgian ancestry, and R169Q, which is thought to be associated with susceptibility to stroke, in the European population [4]. We identified 14 mutations, and 7 of which have not been reported among non-Chinese patients. These mutations are distributed throughout the gene, without a preferential location in specific domains. None of our patients carried the G47R mutation. Only one patient with the R169Q mutation was identified but without hematologic or neurological manifestations. Chinese DADA2 patients may have different mutation frequencies and genotypes. Furthermore, it remains to be elaborated whether DADA2 patients of different ethnicities share similar genotype-phenotype correlations.

There is increasing evidence that there are diverse presentations of ADA2 deficiency, even among patients with identical mutations [16]. Two subjects in our series had growth retardation compared to the 1-year-old standard, which has not been previously described in children with DADA2. The two cases were both early-onset, before 6 months, but these children were born with average weight and length and were cared for with adequate feeding. It remains to be established whether DADA2 affects the growth of children, or whether growth retardation is a new phenotype. Nonetheless, growth assessment should be taken into account when assessing pediatric patients. According to previous reports, the neurological manifestations of the central and peripheral nervous system represent permanent damage and dysfunction. Unilateral hearing loss can be observed during disease flares [17]. Three of our patients had neurologic involvement accompanied by ophthalmological dysfunction, such as eye external oblique and vision diminution, but all signs were reversed by suitable treatment. Early recognition of central nervous system dysfunction signs and appropriate therapy are essential for children with DADA2, because there might be a chance to avoid permanent sensory impairment.

Specific clinical features, such as pure red cell aplasia and bone marrow failure, were reported to correlate with the lower level of ADA2 enzyme activity [7]. But our patients with the level lower than 1.0 U/L did not completely coincide with features, and none of them presented with hepatosplenomegaly or TBNK subset deficiency. This may have been influenced by the age of onset, race, and disease course. Other symptoms, such as recurrent fever, livedo reticularis, necrosis, and arthritis, are consistent with cases reported previously [3, 15]. Chinese DADA2 patients have higher rates of fever and abdominal pain than non-Chinese DADA2 patients. Due to the small number of DADA2 in China, the number of cases at home and abroad is very different; thus, the reliability of the comparison results might be insufficient, and it is necessary to expand the sample size further.

Steroids are the mainstay of treatment, however, flares of inflammation and vasculitis upon tapering often occur. Immunosuppressive therapies, including azathioprine, cyclophosphamide, and methotrexate, have all been used, though little success in severe cases has been reported [3]. Except for P1, the patients received steroids together with immunosuppressants as initial treatment, and two achieved complete responses. This may suggest that immunosuppressive therapies can still provide good results in some cases. Anti-TNF agents are the currently preferred treatment based on the positive effect, in particular, in preventing stroke [6, 18]. Regardless, there is little mechanistic evidence for particular treatment choices. Recently, it was found that in the absence of ADA2, the formation of neutrophil extracellular traps could be triggered, resulting in the production of TNF-α [19]. This mechanism may explain the basis of anti-TNF therapy. Three of our patients received anti-TNF therapy; only one of them had a significantly elevated level of TNF-α before, and all showed a good response, with no stroke occurring after initiation. The only patient missing anti-TNF initiation, who had the lowest level of TNF-α, unfortunately had a stroke 6 months after onset. It is unclear whether the level of TNF-α matches the prognosis of DADA2.

In contrast to anti-TNF therapy, the anti-IL-6 agent tocilizumab seems to fail. Several studies have suggested that high serum IL-6 levels can be observed in DADA2 patients, and the use of tocilizumab has been reported to control recurrent fever [18, 20, 21]. Furthermore, it was reported that tocilizumab induced full remission in a DADA2 patient with an idiopathic multicentric Castleman disease-like phenotype [22]. Two of our patients with markedly increased serum IL-6 levels received tocilizumab. Unfortunately, the remission of both lasted for no more than half a year, and both cases relapsed. There are reports of three other patients with recurrent stroke while on tocilizumab [18, 23, 24]. The findings for our patients support that DADA2 patients without a Castleman disease-like phenotype are less responsive to anti-IL-6 therapy.

In some DADA2 patients, the hematological phenotype may be an accompanying feature., including bone marrow failure and pure red cell aplasia. The commonly used immunosuppressive drugs and TNF-α inhibitors may not reverse the hematological manifestation [7, 25], though HSCT has been considered a treatment, specifically for patients with this phenotype [26]. Indeed, one of our patients underwent HSCT. Before that, treatments with steroids, tocilizumab, and anticoagulant therapy were attempted (P7), but all proved unsuccessful. Although her HSCT was successful, this patient did not present with severe hematological defects such as pure red cell aplasia or bone marrow failure. In addition, anti-TNF therapy, which is preferential for DADA2 patients with vasculitis (as for P7), was missed. In general, the negative side effects of the HSCT should not be neglected, and the indication for the particular treatment choice should be considered twice.

### Limitation

The present study has several limitations. First, the present study was from a single center, and the number of patients was small. Further studies should be conducted with multi-center collaboration. As no consensus about the reference of ADA2 enzyme activity had been published, we did refer their parents, who are the carrier of ADA2 mutations, as controls.

## Conclusion

This study includes the largest number of Chinese DADA2 patients to date. Fourteen mutations in ADA2 were identified, with 7 sites not being reported in other countries. It is important to screen the patients with specific clinical features of DADA2. We suggest that Chinese DADA2 patients may have different genotypes. Growth retardation might be a new phenotype among DADA2 patients. Anti-IL-6 therapy may fail to achieve disease remission.

## Data Availability

All data generated or analyzed during this study are included in this published article.

## Abbreviations

ADA2: Adenosine deaminase 2
DADA2: Deficiency of adenosine deaminase 2
CECR1: Cat eye syndrome chromosome region, candidate 1
CRP: C-reactive protein
ESR: Erythrocyte sedimentation rate
Ig: Immunoglobulin
IL: Interleukin
CS: Corticosteroid
MTX: Methotrexate
MMF: Mycophenolate mofetil
TCZ: Tocilizumab
TNF: Tumor necrosis factor
HSCT: Hematopoietic stem cell transplantation
